# Supplementary figures and images for: Characterization of Extraintestinal Pathogenic Escherichia coli Strains Causing Canine Pneumonia in China: Antibiotic Resistance, Virulence Genes, and Sequence Typing
Source: Vet Sci. 2024 Oct 10;11(10):491. doi: 10.3390/vetsci11100491 (PMC11512281; doi:10.3390/vetsci11100491)

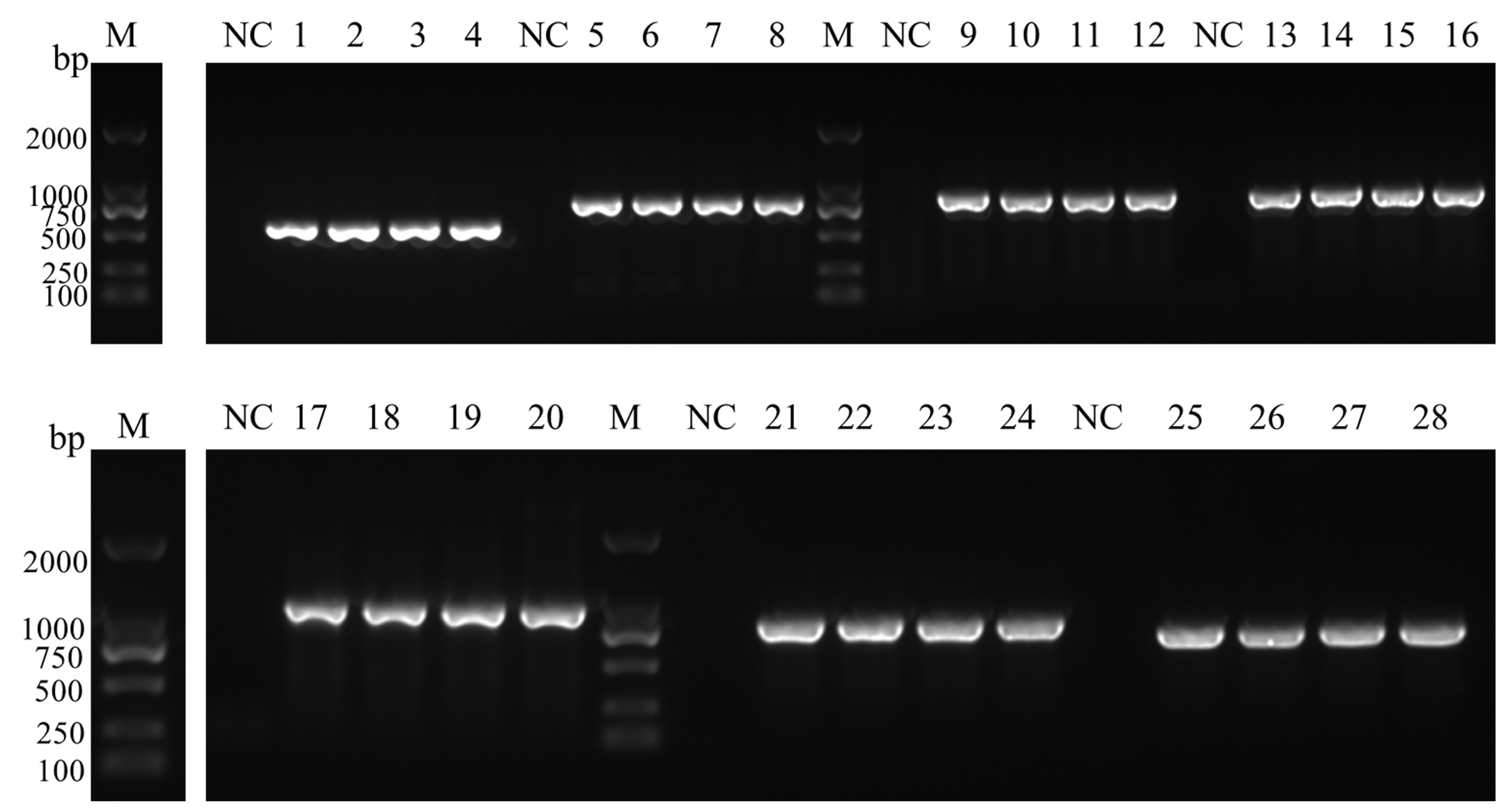

Supplement: Supplementary file 1 [file vetsci-11-00491-s001.zip › Figure S3.png]

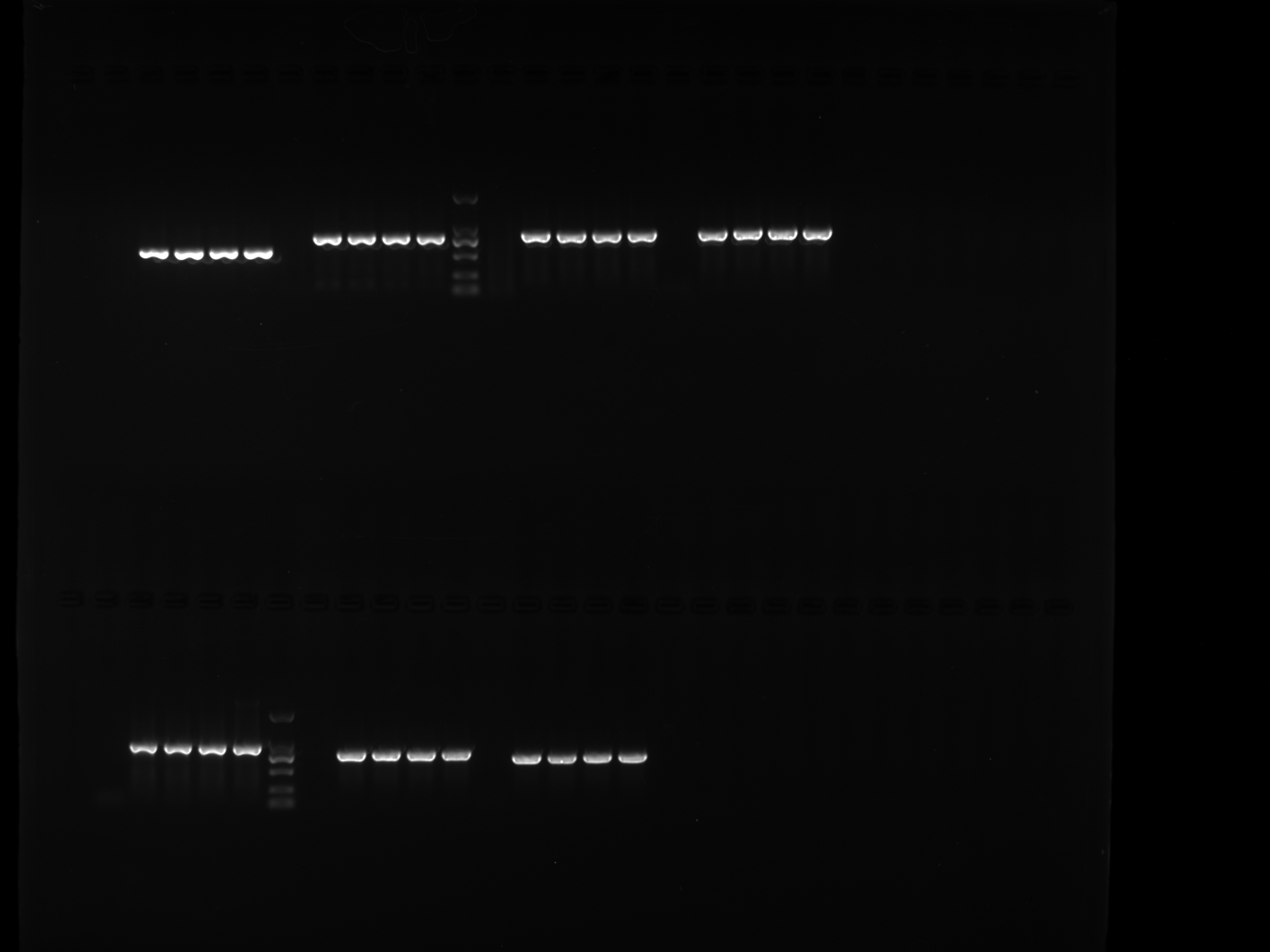

Supplement: Supplementary file 1 [file vetsci-11-00491-s001.zip › Figure S3.tif]

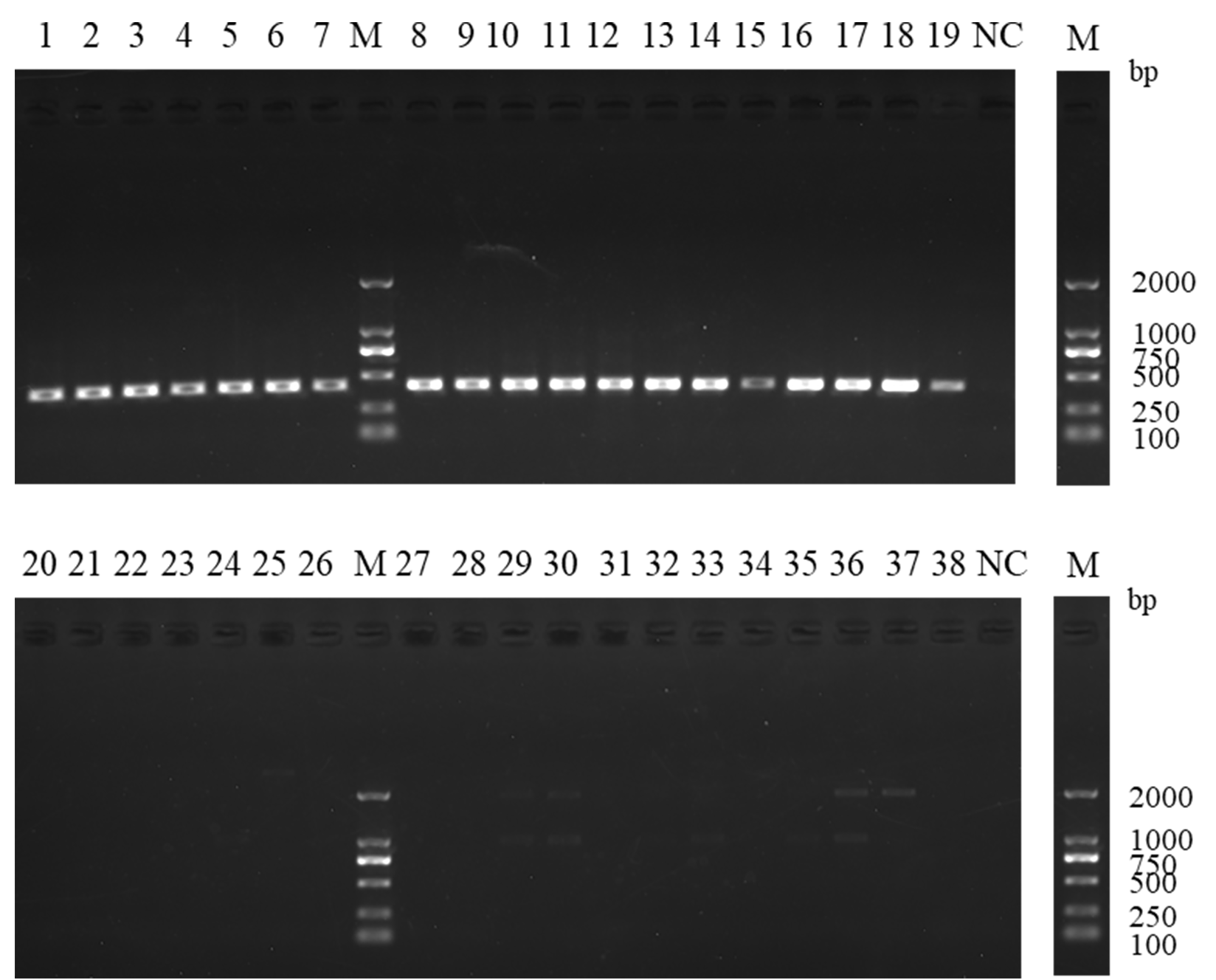

Supplement: Supplementary file 1 [file vetsci-11-00491-s001.zip › Figure S4.png]

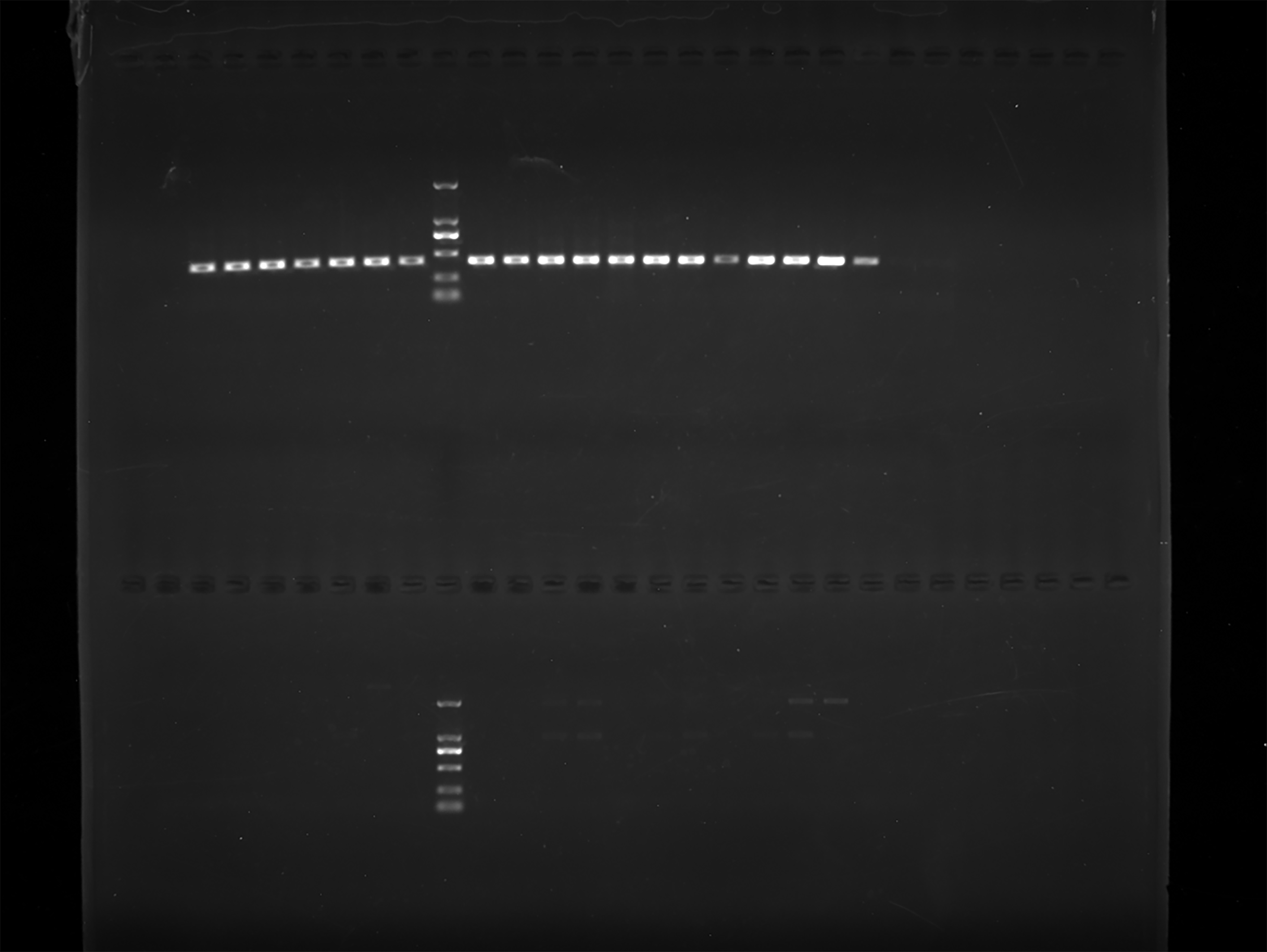

Supplement: Supplementary file 1 [file vetsci-11-00491-s001.zip › Figure S4.tif]

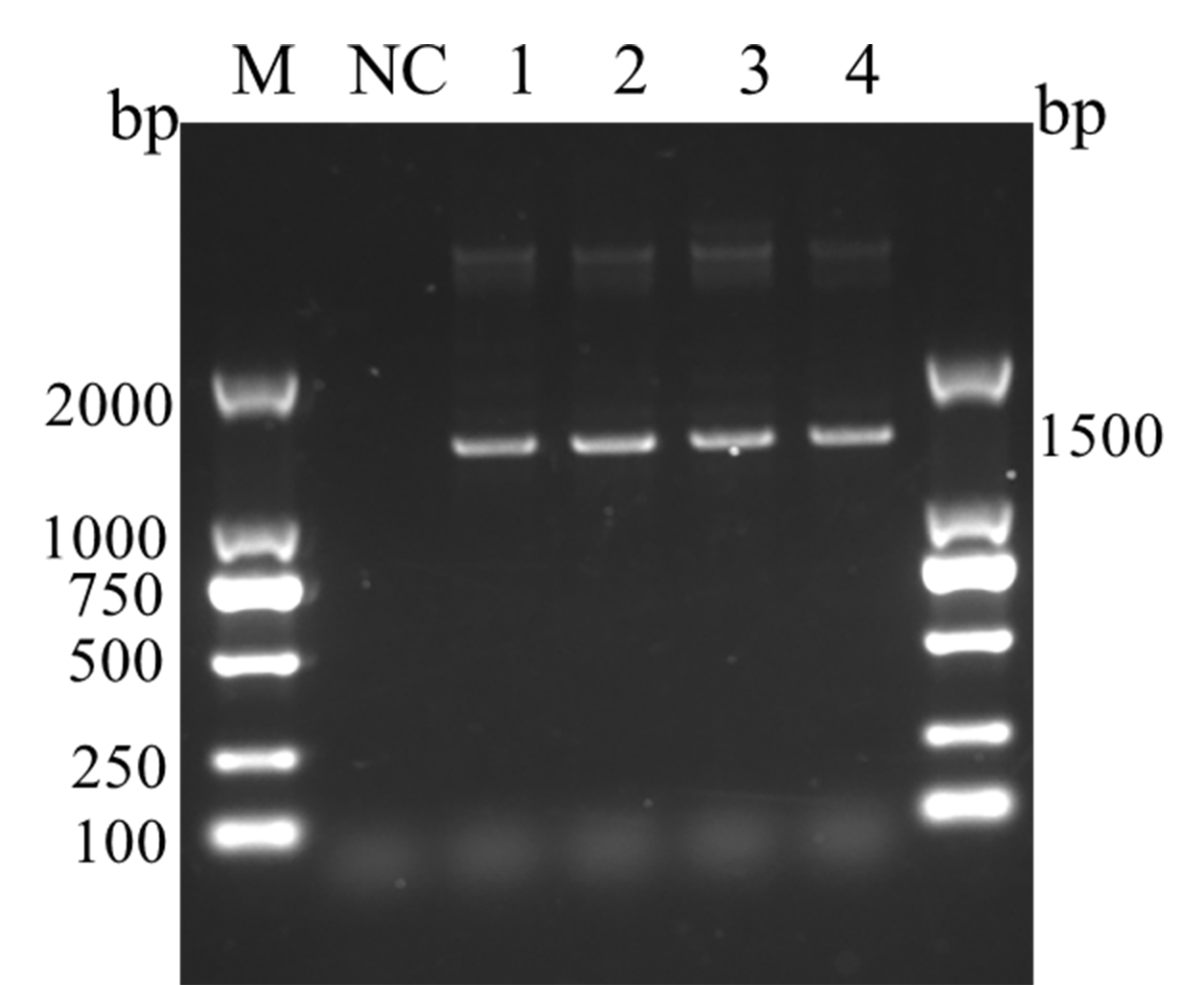

Supplement: Supplementary file 1 [file vetsci-11-00491-s001.zip › Figure S1.png]

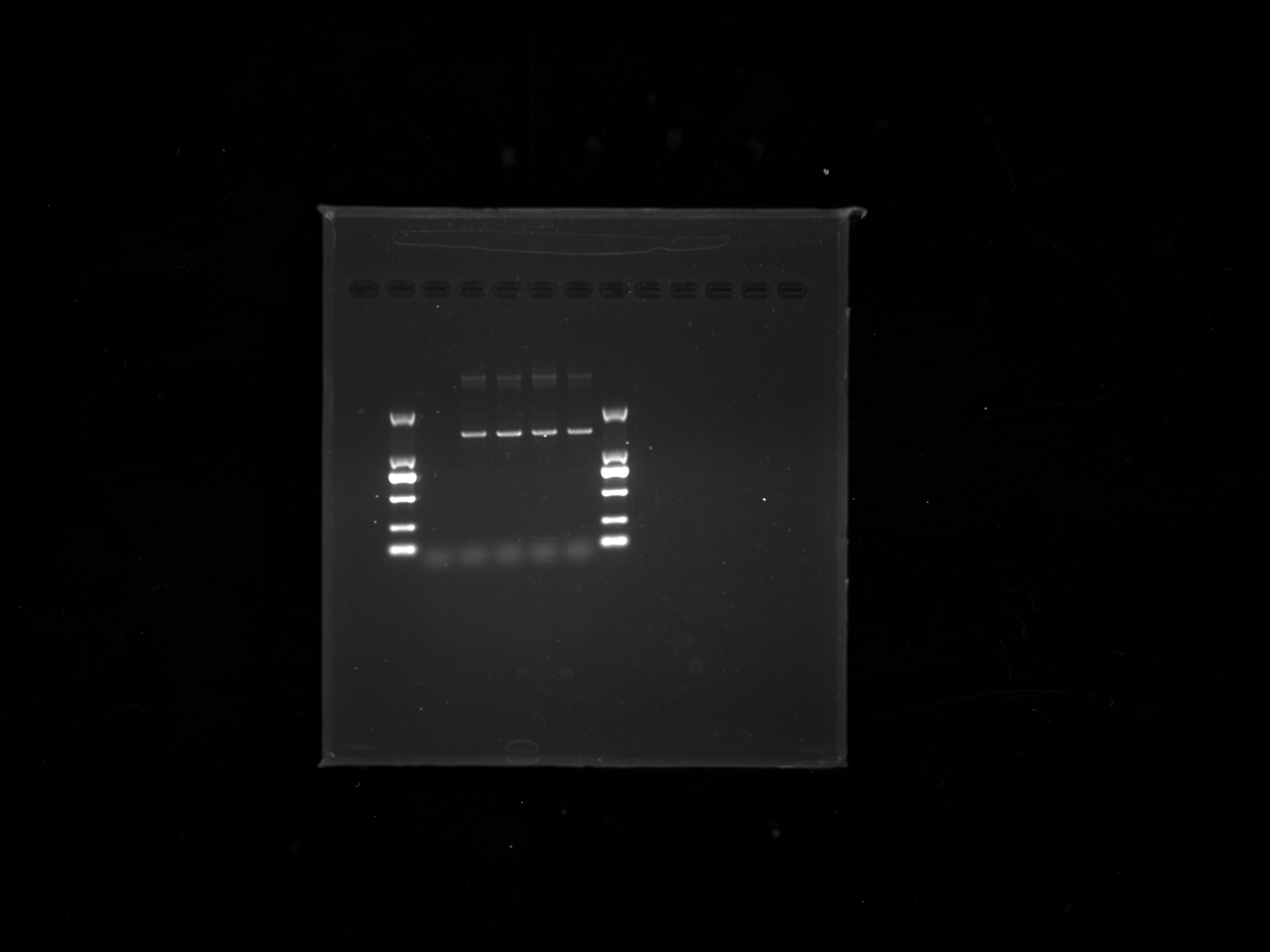

Supplement: Supplementary file 1 [file vetsci-11-00491-s001.zip › Figure S1.tif]

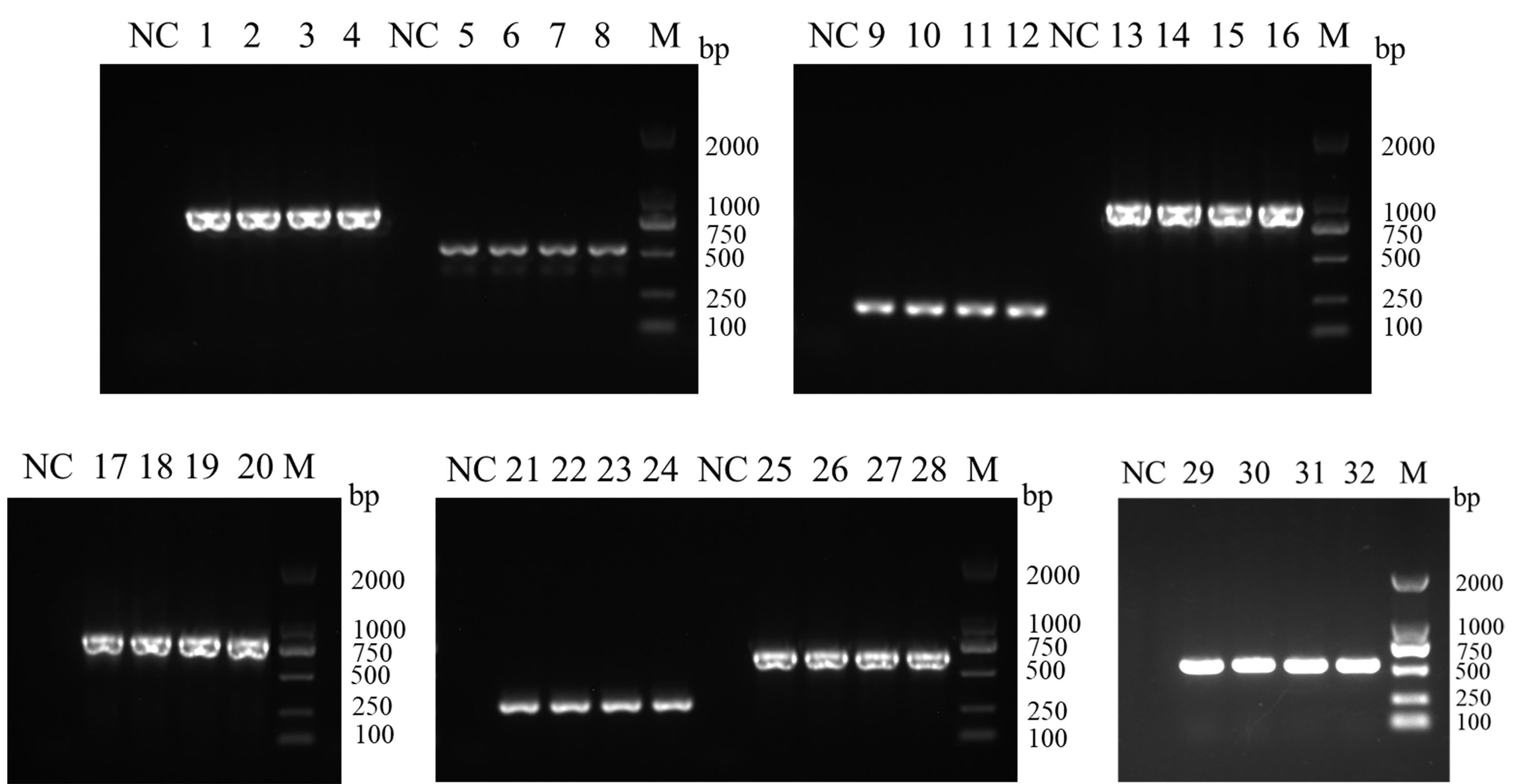

Supplement: Supplementary file 1 [file vetsci-11-00491-s001.zip › Figure S2.png]

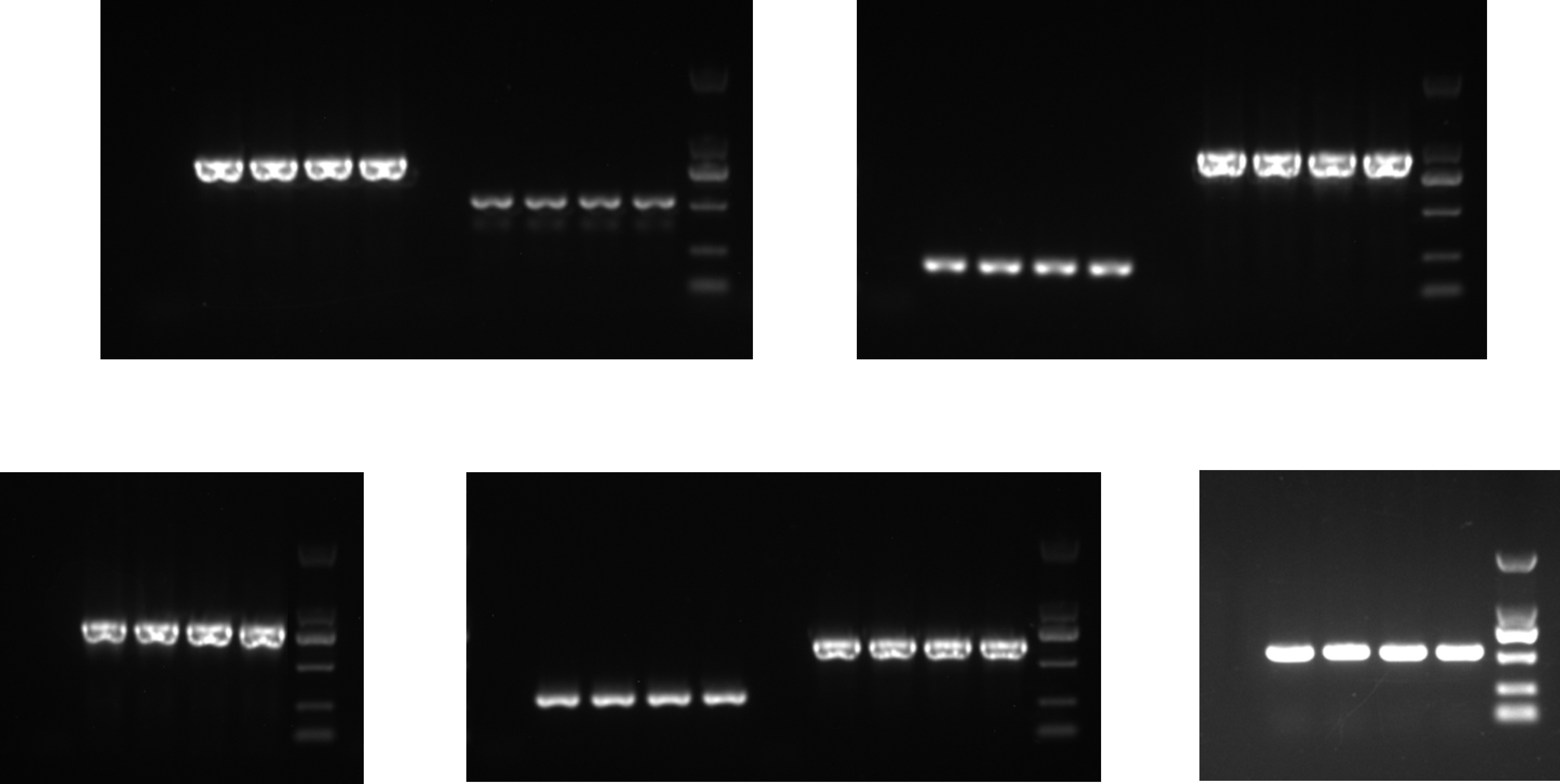

Supplement: Supplementary file 1 [file vetsci-11-00491-s001.zip › Figure S2.tif]
